# Diverse phosphorylation patterns of B cell receptor-associated signaling in naïve and memory human B cells revealed by phosphoflow, a powerful technique to study signaling at the single cell level

**DOI:** 10.3389/fcimb.2012.00128

**Published:** 2012-10-17

**Authors:** Franklin R. Toapanta, Paula J. Bernal, Marcelo B. Sztein

**Affiliations:** ^1^Department of Medicine, Center for Vaccine Development, University of MarylandBaltimore, MD, USA; ^2^Department of Pediatrics, Center for Vaccine Development, University of MarylandBaltimore, MD, USA

**Keywords:** naïve B cells, IgM memory B cells, cell signaling, phosphoflow, fluorescent-cell barcoding, *Salmonella* Typhi, *Streptococcus pneumoniae*, vaccines

## Abstract

Following interaction with cognate antigens, B cells undergo cell activation, proliferation, and differentiation. Ligation of the B cell receptor (BCR) leads to the phosphorylation of BCR-associated signaling proteins within minutes of antigen binding, a process with profound consequences for the fate of the cells and development of effector immunity. Phosphoflow allows a rapid evaluation of various signaling pathways in complex heterogenous cell subsets. This novel technique was used in combination with multi-chromatic flow cytometry (FC) and fluorescent-cell barcoding (FCB) to study phosphorylation of BCR-associated signaling pathways in naïve and memory human B cell subsets. Proteins of the initiation (Syk), propagation (Btk, Akt), and integration (p38MAPK and Erk1/2) signaling units were studied. Switched memory (Sm) CD27+ and Sm CD27− phosphorylation patterns were similar when stimulated with anti-IgA or -IgG. In contrast, naïve and unswitched memory (Um) cells showed significant differences following IgM stimulation. Enhanced phosphorylation of Syk was observed in Um cells, suggesting a lower activation threshold. This is likely the result of higher amounts of IgM on the cell surface, higher pan-Syk levels, and enhanced susceptibility to phosphatase inhibition. All other signaling proteins evaluated also showed some degree of enhanced phosphorylation in Um cells. Furthermore, both the phospholipase C-γ2 (PLC-γ2) and phosphatidylinositol 3-kinase (PI3K) pathways were activated in Um cells, while only the PI3K pathway was activated on naïve cells. Um cells were the only ones that activated signaling pathways when stimulated with fluorescently labeled *S*. Typhi and *S. pneumoniae*. Finally, simultaneous evaluation of signaling proteins at the single cell level (multiphosphorylated cells) revealed that interaction with gram positive and negative bacteria resulted in complex and diverse signaling patterns. Phosphoflow holds great potential to accelerate vaccine development by identifying signaling profiles in good/poor responders.

## Introduction

The main receptor on the surface of B cells is the B cell receptor (BCR), which is composed of a membrane immunoglobulin (Ig) and an Igα/Igβ heterodimer (Defranco, 1997; Schamel and Reth, 2000). The BCR is responsible for antigen binding and signaling and plays a central role in determining the fate of B cells during their developmental process and later when naïve cells encounter foreign antigens. In naïve B cells, ligation of the BCR by cognate antigens initiates a series of responses/signal cascades that will induce cells to proliferate and differentiate, which will ultimately lead to the production of antibodies specific for the pathogenic microorganism (Benschop and Cambier, 1999). Therefore, signaling pathways associated with the BCR are crucial for B cell development, activation, proliferation, differentiation (e.g., memory and plasma B cells) and apoptosis (Defranco, 1997; Benschop and Cambier, 1999; Dal Porto et al., 2004).

The signaling pathways associated with the BCR are complex and involve multiple molecules which can be divided into small units for simplicity: (1) signal initiation unit, which involves early events associated with the BCR-complex itself; (2) signal propagation unit, which involves signal amplification and diversification of the BCR, via key effector and adaptor molecules that lead to downstream effectors and the production of second messenger molecules; (3) signal integration unit, which involves the pathways by which these downstream effectors/second messengers regulate transcription factor activation and therefore gene expression [reviewed in: Dal Porto et al. (2004); Kurosaki (1999, 2000, 2011); Gold (2002); Defranco (1997)]. In the signal initiation unit, engagement of BCR by antigens allows tyrosine phosphorylation of the intracellular Igα/Igβ immunoreceptor tyrosine-based activation motifs (ITAMs) by Src-family kinases, such as Lyn, leading in turn to the activation of the spleen tyrosine kinase (Syk) (Takata et al., 1994; Lankester and Van Lier, 1996; Defranco, 1997; Kurosaki, 1999; Dal Porto et al., 2004). This initiates assembly of the signalosome (signal propagation unit), composed of a variety of intracellular signaling molecules, such as Vav, Btk, phosphatidylinositol 3-kinase (PI3K), and phospholipase C-γ2 (PLC-γ2) (Kurosaki, 1999; Dal Porto et al., 2004; Scharenberg et al., 2007), as well as adaptor proteins, such as B cell linker (BLNK) and B cell adaptor for PI3K (BCAP) (Goitsuka et al., 1998; Okada et al., 2000). These adaptors proteins have served to identify two main BCR-associated signaling cascades: (1) PLC-γ2 and (2) PI3K pathways. In the PLC-γ2 pathway, BCR stimulation leads to phosphorylation of Igα/Igβ ITAMs, followed by Syk recruitment and activation (phosphorylation). Activated Syk, phosphorylates BLNK (Ishiai et al., 1999a,b), allowing the binding of PLC-γ2 and Btk. PLC-γ2 becomes activated by Syk and Btk to produce diacylglycerol (DAG) and inositol-1,4,5-triphosphate (IP3) from phosphatidylinositol-4,5-bisphosphate (PIP2), leading to release of intracellular Ca^2+^, activation of Ca^2+^-release activated Ca^2+^ channels (CRAC) and extracellular Ca^2+^ influx (Baba et al., 2001; Tomlinson et al., 2001). In the PI3K pathway, after BCR stimulation, CD19 is phosphorylated by Lyn, and BCAP is phosphorylated by Syk and Btk. Phosphorylated CD19 or BCAP activates PI3K to produce phosphatidylinositol (3,4,5)-trisphosphate (PIP3) from PIP2, and the resultant PIP3 transmits signals downstream, including the activation of Akt and therefore activation of the cell survival pathway (Okada et al., 2000; Koyasu, 2003; Cuni et al., 2004). An important aspect of the BCR signaling is the connection between intracellular signaling pathways with activation of transcription factors and regulation of gene expression in the nucleus (signal integration unit). A primary integration point of BCR signaling is the mitogen activated protein kinase (MAPK) family, consisting of three members: extracellular signal-regulated kinase (ERK), c-Jun N-terminal kinase (JNK/SAPK), and p38MAPK (Dong et al., 2002; Johnson and Lapadat, 2002). Following activation, these kinases phosphorylate different sets of transcription factors including (1) E twenty-six (ETS)-like transcription factor 1 (Elk-1) and c-Myc by ERK, (2) c-Jun and activating transcription factor 2 (ATF-2) by JNK, and (3) ATF-2 and myc-associated factor X (MAX) by p38MAPK. The BCR is connected to these “downstream” effectors by a “phosphorelay” system of cytoplasmic kinases which phosphorylate and regulate each other (Johnson and Lapadat, 2002). In summary, BCR ligation leads to activation of its signaling pathways, which triggers a variety of intracellular processes and concludes with phenotypic and genotypic modifications defining the B cell response to the triggering stimulus.

Immunological memory is a very effective mechanism developed by the immune system to ensure rapid protection against infections (Tangye and Tarlinton, 2009) and critical for long-term protection elicited by vaccination (Levine and Sztein, 2004). The phenotypic analysis of human B cell subpopulations by flow cytometry (FC) using the IgD/CD27 classification scheme, currently accepted by most investigators, leads to the identification of four populations: (1) naïve [IgD+CD27−], (2) unswitched memory (Um) [CD27+IgD+], (3) switched memory CD27+ (Sm CD27+) [CD27+IgD−], and (4) switched memory CD27− (Sm CD27−) [CD27−IgD−] B cells (Sanz et al., 2008; Pauli et al., 2011). Some of these populations have been associated with pathogenic conditions. For example, the recently described Sm CD27− B cells are more abundant in patients with active SLE and in patients with RVS infection (Fecteau et al., 2006; Wei et al., 2007; Sanz et al., 2008). Um B cells, also known as memory IgM B cells, are important for protection against encapsulated bacteria [e.g., *S. pneumoniae* and *N. gonorrhea* (So et al., 2012; Kruetzmann et al., 2003)] and involved in T-cell independent immune responses (Weller et al., 2004). Interestingly, these cells have been suggested to be a different population of innate or naïve B cells, but not a true memory B (B_M_) population. Therefore, despite the progress made in understanding naïve and B_M_ cell subpopulations, considerable gaps in knowledge remain. It has become clear that there is a big heterogeneity in the B cell compartment and our pre-conceived notions of function, as well as cellular and anatomical origin, need to be explored in further detail.

The study of BCR-associated signaling pathways has grown exponentially during the last decade, but still relies largely on traditional biochemical methods (e.g., western blots, ELISA). Additionally, most of the published literature relies on established cell lines [e.g., DT40 (Takata et al., 1995; Lindvall and Islam, 2002)], cell transfected with different B cell signaling molecules [e.g., drosophila S2 (Rolli et al., 2002)], or mouse models (Su et al., 2002; Srinivasan et al., 2009; Young et al., 2009; Woyach et al., 2012). Despite the usefulness of these methodologies and models, there are limitations, including the difficulty on translating the results to humans and the study phosphorylation patterns at the single cell level which will enable the investigation of signaling pathways in individual cell subsets. Due to relatively large number of cells required to perform traditional assays and the difficulty to sort cells without altering their signaling profile, the study of BCR-associated signaling pathways in human B cell subpopulations and individual cells has proven challenging. The use of fluorescently labeled monoclonal antibodies for specific phosphorylated epitopes and the development of advanced multichromatic FC techniques have allowed the development of a new technology (phosphoflow) for the study of signaling pathways in primary human cells (Krutzik and Nolan, 2003; Irish et al., 2006; Schulz et al., 2007; Galligan et al., 2009; Krutzik et al., 2011b). This novel technology, in spite of still being in development, has already been used in clinical studies, particularly in blood cancer research (e.g., lymphomas), to identify basic aspects of the cell biology of cancerous cells and susceptibility to chemotherapeutic agents (Irish et al., 2004; Nolan, 2006; Chen et al., 2008; Galligan et al., 2009). However, the potential use of this technology to understand basic aspects of B cell biology in normal and pathologic conditions is enormous and has yet to be realized. To begin exploring the power of this technology to study basic biology and its potential usefulness to accelerate vaccine development, we developed a staining technique that allows identification of naïve and memory cells using the IgD/CD27 classification scheme and explored the differences in activation of BCR-associated signaling pathways in different B cell subpopulations in peripheral blood mononuclear cells (PBMC) of healthy volunteers. Our approach involves the simultaneous measurement of various phosphoproteins and, to multiplex the technique, we coupled the phosphoflow assay with fluorescent-cell barcoding (FCB) (Krutzik and Nolan, 2006; Krutzik et al., 2011a). The results demonstrate the feasibility of studying cell signaling pathways in B cell subpopulations by FC at the single cell level and highlights important differences in the BCR-associated signaling pathways among these cell subpopulations. Furthermore, the use of fluorescently labeled bacteria led to the identification of B cell subpopulations that interact with these bacteria and demonstrated differences in the signaling profile triggered by gram positive and gram negative microorganism.

## Materials and methods

### Human volunteers and isolation of peripheral blood mononuclear cells (PBMC)

PBMC collected from 8 healthy adult volunteers, recruited from the Baltimore-Washington area and University of Maryland, Baltimore campus, were used in this study. Written informed consent was obtained from all volunteers and the procedures approved by the University of Maryland, Baltimore IRB. PBMC were isolated immediately after blood draws by density gradient centrifugation and cryopreserved in liquid nitrogen following standard techniques (McArthur and Sztein, 2012).

### Cell surface staining for flow cytometry

Cryopreserved PBMC were thawed, allowed to rest overnight in complete media [RMPI (Gibco, NY, USA) supplemented with 10% fetal bovine serum (FBS) (Gemini Bioproducts, West Sacramento, CA), 2 mM L-glutamine (Gibco, Grand Island, NY, USA), 1× non-essential amino acids (Gibco, Grand Island, NY, USA), 10 mM HEPES (Gibco, Grand Island, NY, USA), 2.5 mM Sodium pyruvate, (Lonza, Walkersville, MD, USA), 100 U/ml Penicillin, 100 ug/ml streptomycin (Sigma-Aldrich, St. Louis, MO, USA), 50 μg/ml Gentamicin (Gibco, Grand Island, NY, USA)] and stained for FC in V-shaped 96-well plates using methods previously described (Toapanta and Ross, 2009; McArthur and Sztein, 2012). Briefly, 1 × 10^6^ cells were plated and stained for viability using a violet-fixable dye (Invitrogen, USA). After two washes with FC staining buffer (4% FCS, 1× phosphate buffered saline (PBS), and 0.02 Sodium Azide) cells were blocked with mouse IgG (25 μl of a 2.0 μg/ml solution in FC buffer) (Meridian Life Sciences, Memphis, TN, USA) and stained with an antibody cocktail prepared in FC buffer. Antibodies used included: IgD-FITC (polyclonal sera goat anti-human; Southern Biotechnologies), IgG-PE (polyclonal sera goat anti-human; Southern Biotechnologies), CD19-ECD (clone:J3-119; Beckman Coulter), IgA-Biotin (polyclonal sera goat anti-human; Southern Biotechnologies), IgD-APC (clone: IA6-2; Becton Dickinson -BD-), CD27-APC-Alexa700 (clone: 1A4CD27; Beckman Coulter), and CD3-Pacific Blue (clone: UCHT1; BD). After washing the cells 2× with FC buffer, Pacific Orange-Streptavidin was added to each well (Invitrogen, USA). Stained cells were fixed with 1% PFA in PBS.

### Labeling of bacteria with Alexa 700 (Ax700)

Wild-type *S*. Typhi strain Ty2 (Deng et al., 2003) was grown in LB broth at 37°C while shaking. *S. pneumoniae* (ATCC 6303) was grown in Brain Heart infusion (37°C, 5% CO_2_). Bacteria cells were grown until an OD of 0.8 (600 nm) was reached. Ten ml of this bacteria culture solution were spin are re-suspended in 1 ml of PBS. Cells were then heat-inactivated for 30 min at 56°C and then transferred to ice cold water for 2–3 min. Bacteria cells were then washed once in 10 ml of PBS (3000 rpm, 4°C, 15 min), the pellet re-suspended in 500 μl of an Ax700-succinimidyl solution (2 μg/ml), and stained for 30 min on ice in the dark. Bacteria cells were then washed 2× with 10 ml of FC buffer and then fixed with 1% paraformaldehyde (PFA) in 1× PBS for 20 min on ice in the dark. Cells were washed 2× (FC buffer) and re-suspended in 1% bovine serum albumin (BSA) at 0.8 OD and stored at 4°C. Appropriate bacteria staining were confirmed by FC using a MoFlo flow cytometer/cell sorter system (Beckman Coulter, USA). To determine the CFU contained in 0.8 OD suspensions, 10-fold dilution of freshly harvested bacteria were plated into LB agar or sheep blood agar plates and cultured overnight (37°C for *S*. Typhi and 37°C, 5% CO_2_ for *S. pneumoniae*).

### Phosphoflow

#### Stimulants used in phosphoflow experiments

Cells were stimulated either with goat anti-human IgM, IgG, or IgA (Southern Biotech, USA) (5 μg/ml) polyclonal sera. A mixture of goat anti-IgM, IgD, IgG, IgA (2.5 μg/ml each) and 6 mM Hydrogen peroxide (H_2_O_2_) was used as a positive stimulation control and 1% BSA in PBS as a negative stimulation control. For experiments involving stimulation with bacteria, 50 μl of 0.8 OD Ax700 labeled *S*. Typhi or *S. pneumoniae* were used (this is equivalent to a MOI of 100 to 1).

#### Cell stimulation

PBMC incubated overnight in complete media at 37°C, 5% CO_2_ were re-suspended in ice cold 1% BSA, transferred to 12 × 17 mm tubes (50 μl containing 5 × 10^5^/sample) and maintained in ice for 15 min. Meanwhile, stimulants (e.g., goat anti-IgM) were prepared freshly in 1% BSA in PBS. Stimulant aliquots (50 μl) were maintained at room temperature and added to the chilled PBMC and immediately transferred to a water bath (37°C) for different time periods. After stimulation, cells were fixed with 25 ul of 10% PFA at room temperature (10–20 min) and washed 2× with FC buffer.

#### Staining and fluorescent-cell barcoding

Fixed cells were stained with anti-human IgD-FITC (Southern Biotech, USA) for 20 min on ice in the dark, washed 2× with FC buffer and permeabilized with 80% ice cold methanol (600 μl) for 20 min at −20°C. In experiments that required FCB, pacific orange-succinimidyl at different concentrations were prepared (80 and 400 μg/ml); 5 ul of each concentration was added to methanol permeabilized cells, followed by 395 ul of 1× PBS. Samples were mixed vigorously and allowed to stain for 45 min on ice in the dark, rehydrated, pooled, and transferred to a fresh 12 × 17 mm tube. Cells were directly rehydrated when FCB was not required. To rehydrate PBMC, samples were washed 2× with FC buffer. Samples were then blocked with human IgG (25 ul of a 1 mg/ml solution in FC buffer) (Sigma-Aldrich, St. Louis, MO, USA) for 10 min and then stained with antibody cocktails in FC buffer for 1 h on ice in the dark. Antibodies in the cocktails included: CD27-PE (clone: L128; BD), pAKT-S473-Biotin (clone: D9E; Cell Signaling Technologies), CD20-PerCP-Cy5.5 (clone: H1; BD), p38MAPK-T180/Y182-Pacific Blue (clone: 36/p38 (pT180/pY182); BD), Syk-Y352-Alexa647 (clone: 17A/P-ZAP70; BD), Erk1/2-T202/Y204-Pacific Blue (clone: 20A; BD), p38MAPK-T180/Y182-PE-Cy7 (clone: 36/p38 (pT180/pY182); BD), and/or Btk-Y551-Alexa647 (clone: BtkY551 & ItkY511; BD). Cells were washed 2× with FC buffer. In panels that needed a secondary staining step, ECD-Streptavidin (Beckman Coulter) was added, cells incubated on ice for 30 min, and washed 2× in FC buffer. Cells were then fixed with 1% PFA in 1× PBS/EDTA and samples collected in a custom LSRII flow cytometer analyzer (Beckton-Dickson, USA). Samples were analyzed using a FlowJo software package (Tree Star, USA). Statistical analysis was performed in GraphPad Prism software (GraphPad Software Inc., USA).

## Results

### Distribution of naïve and B_M_ cell populations in human PBMC and cell surface immunoglobulin (Ig) isotype expression

**Figure 1:**
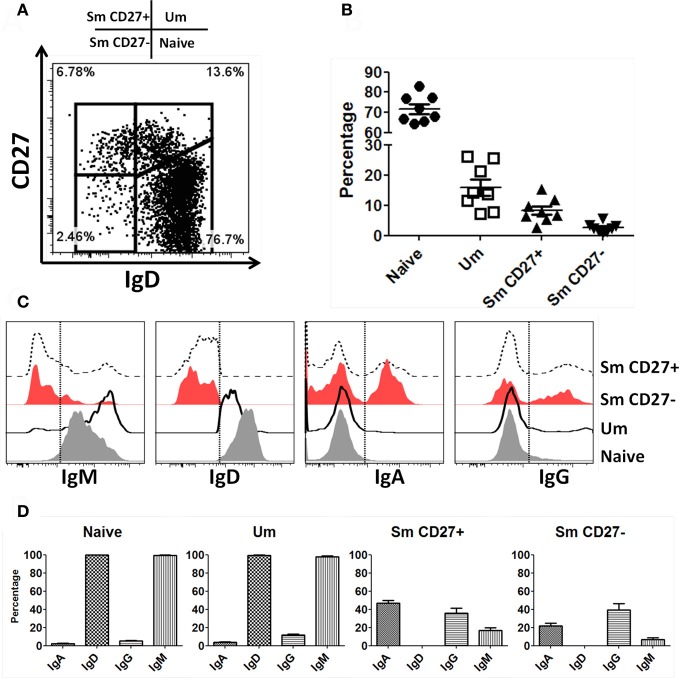
**Naïve and B_M_ cell subpopulations defined by IgD and CD27 markers in PBMC.** Four B cell (CD19+ CD3−) subpopulations are defined by expression of IgD and CD27: Naïve (IgD+ CD27−), Um (IgD+ CD27+), Sm CD27+ (IgD− CD27+), and Sm CD27− (IgD− CD27−) **(A)**. The percentages of these B-cell subpopulations showed little variation in 8 healthy *adult* volunteers **(B)**. An example of surface Ig isotype (IgM, IgD, IgA, and IgG) distribution in B cell subpopulations in a representative volunteer is shown **(C)**. Dotted lines indicate where the gates were set to determine the percentage of each Ig class. **(D)** Compiled data of the percentages of the Ig isotypes on the surface of each B-cell subpopulation (*n* = 8).

B cells were identified in PBMC of healthy volunteers as CD19+ and CD3− cells. Subsequently, naïve and B_M_ subpopulations were defined using IgD and CD27 as previously reported (Sanz et al., 2008). These markers show four distinct populations of B cells: naïve (IgD+ CD27−), Um (IgD+ CD27+), Sm CD27+ (IgD− CD27+) and Sm CD27− (IgD− CD27−) (Figure 1A). In PBMC from 8 screened volunteers, naïve B cells were the most abundant population (mean 71.7%; SE ± 2.4), followed by Um (mean 15.9%; SE ± 2.7), Sm CD27+ (mean 8.2%; SE ± 1.4) and Sm CD27− (mean 2.5%; SE ± 0.6) (Figure 1B). Expression of surface Ig isotypes was evaluated within each of these subpopulations (Figures 1C,D). Naïve as well as Um cells expressed both IgM and IgD, while Sm CD27+ and Sm CD27− B cells expressed mainly IgA and IgG. Interestingly, a small percentage of Sm CD27+ cells also expressed IgM (mean 17%; SE ± 3) (Figures 1C,D).

### Differential expression of IgM and IgD on the surface of naïve and Um B cells

**Figure 2:**
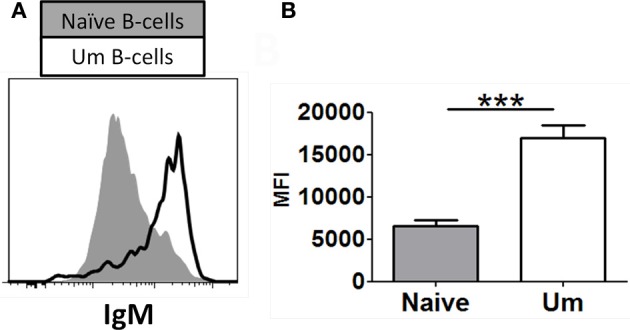
**Naïve and Um B cells show different frequency of IgM molecules on the cell surface.** Um B cells (black line histogram) show a higher IgM mean fluorescence intensity (MFI) on their surface than naïve B cells (gray closed histogram) as shown in this representative volunteer **(A)**. Compiled data from 8 healthy adults **(B)**. ^***^p = 0.0001.

As described above, similar percentages of naïve and Um B cells co-expressed IgM and IgD (Figure 1D). However, Um expressed a higher amount of IgM molecules/cell than naïve B cells (Figures 1C, 2A,B), as determined by mean fluorescent intensity (MFI). These differences were statistically significant (*p* < 0.0001) (Figure 2B). On average Um B cells expressed 2.6-fold more IgM than naïve B cells (Figure 2B).

### Naïve and Um B cells show different Syk phosphorylation intensity patterns following BCR stimulation

**Figure 3:**
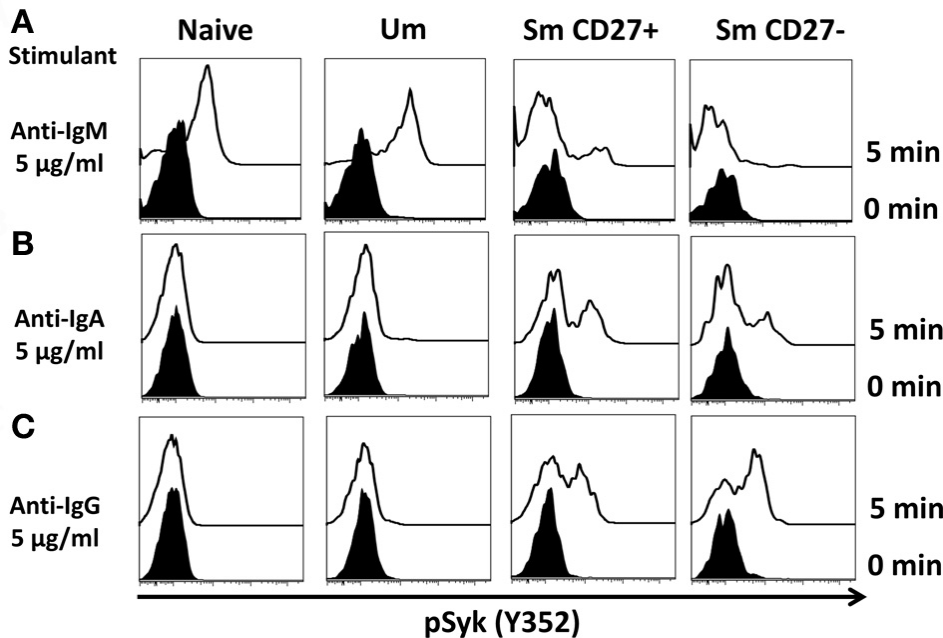
**Anti-IgM induces specific Syk(Y352) phosphorylation in naïve and Um B cells.** Only Um and Naïve B cells, which co-express IgM and IgD, showed robust Syk phosphorylation after PBMC stimulation with anti-IgM **(A)**. Interestingly, a small peak was detected in Sm CD27+ cells, which probably corresponds to IgM cells present in Sm CD27+ cells (Figure 1D). No phosphorylation of Syk was detected in Sm CD27− B cells **(A)**. PBMC were also stimulated with anti-IgA and -IgG (**B** and **C**, respectively). Syk phosphorylation was detected in Sm CD27+ and Sm CD27−, but not in naïve and Um B cells (**B** and **C**).

**Figure 4:**
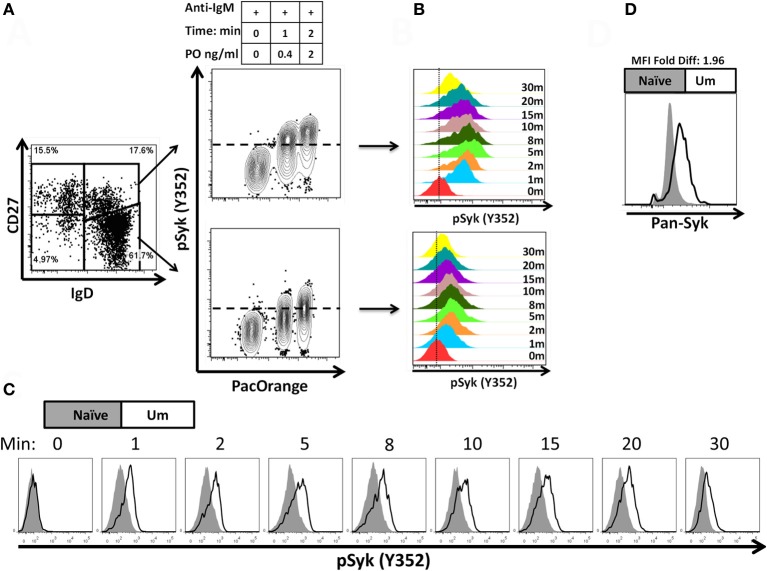
**Fluorescent-cell barcoding (FCB) and time course of Syk phosphorylation in naïve and Um B cells.** FCB was introduced in the pacific orange channel (0, 0.4, and 2 μg/ml) to enable assay multiplexing of 3 samples at a time (see details in “Materials and Methods”) and collected samples were deconvoluted for analysis **(A)**. Time course experiments of PBMC stimulated with anti-IgM showed that both, Um and naïve cells phosphorylated Syk after BCR stimulation **(B)**; however, Um cells showed stronger phosphorylation than naïve cells **(C)**. Higher levels of total Syk molecules are present in Um (black line histogram) than in naïve (gray histogram) B cells **(D)**. Data is shown for one representative volunteer.

**Figure A1:**
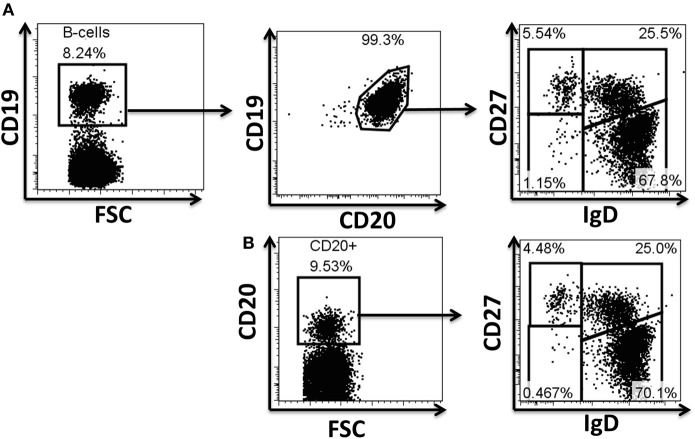
**Evaluation of antibodies for phosphoflow in naïve and B_M_ cell subpopulations.** Example of surface staining for identification of B cells and their subpopulations. CD19 and CD20 are typically used to define B cell populations **(A)**. Several monoclonal antibodies were evaluated to define B cells, as well as naïve and memory B cell subpopulations during the optimization of the staining technique for phosphoflow. The CD19 marker is lost when cells are treated for phosphoprotein determinations; however, CD20 (H1 clone) defines these cells appropriately and therefore was subsequently used in phosphoflow assays **(B)**. Polyclonal anti-IgD sera (goat anti-human) used in a two step staining technique (see “Materials and Methods” for details) provided appropriate resolution of naïve and memory B cell subpopulations for phosphoflow staining **(B)**. FSC, forward scatter.

A staining technique that allowed appropriate resolution of naïve and B_M_ cell subpopulations in conditions suitable to assay phosphorylation of signaling proteins was developed (see “Appendix Figure A1 and Materials and Methods”). To investigate whether this optimized staining technique allowed the evaluation of phosphoproteins associated with the BCR, overnight rested PBMC were stimulated with goat F (ab')2 anti-human IgM (5 μg/ml) for 5 min. Phosphorylation of Syk (pSyk—Y352), one of the phosphotyrosine kinases (PTK) associated with the BCR was then measured in naïve and Um B cells. As expected, Sm CD27+ and Sm CD27− B cells, most of which are IgM negative (Figures 1C,D), did not show significant pSyk (Figure 3A). To further validate this assay, PBMC were stimulated with anti-IgG and anti-IgA antibodies [goat F (ab')2 anti-human IgG or IgA; 5 μg/ml; 5 min]. Under these conditions pSyk was detected in Sm CD27+ and Sm CD27− cells, but not in naïve and Um B cells (Figures 3B,C). In subsequent experiments, FCB, which provides a unique fluorescent signature to cells in independent samples and allows flow cytometric multiplexing, was incorporated in the phosphoflow assay (Figure 4A). FCB enabled the simultaneous measurement of multiple time points in a single tube and provided additional consistency in the staining. Fluorescent-cell barcoded samples were later deconvoluted for analysis (Figure 4B). Syk phosphorylation (Y352) was examined in PBMC stimulated with anti-IgM at various time points (0, 1, 2, 5, 8, 10, 15, 20, and 30 min) (Figure 4C). Similar levels of pSyk were detected in resting naïve and Um B cells (Figure 4C—0 min). Phosphorylation was detected as early as 1 min after anti-IgM stimulation and the intensity of pSyk (MFI) was higher in Um than in naïve B cells at every time point measured. Furthermore, Um B cells remained phosphorylated for a longer period of time (Figure 4C). In order to determine if the differences in Syk phosphorylation were also due to dissimilar levels of this protein, total levels of Syk were evaluated. Um B cells had higher amounts (2-fold) of pan-Syk (phosphorylated and non-phosphorylated) than naïve B cells (Figure 4D).

### Diverse signaling activation pathways in naïve and Um B cells

**Figure 5:**
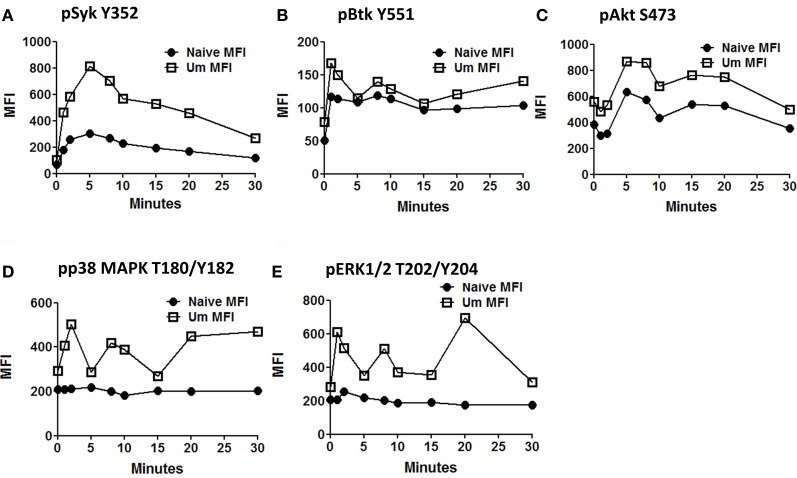
**Time course of signaling proteins and pathways associated with the BCR in naïve and Um B cells.** Two multicolor panels were used to explore simultaneous phosphorylation of signaling proteins and pathways associated with the BCR at various time points. The data shown is from one representative volunteer and is presented as mean fluorescence intensity (MFI). Um demonstrated enhanced phosphorylation potential for all the phosphoproteins assayed compared to naïve B cells (**A** to **E**) following anti-IgM stimulation. Interestingly, Um and naïve B cells demonstrated phosphorylation of Syk, Btk, and Akt (**A,B,** and **C**), which suggest that the PI3K pathway is activated in both cell populations. However, p38MAPK and pERK1/2 (**D** and **E**) are phosphorylated only in the Um subset, suggesting that the PLC-γ2 pathway is also activated in these cells. Additionally, different phosphoproteins have different kinetics. For example, the peak of Syk phosphorylation was detected at 5 min after stimulation **(A)**, and then slowly declined; meanwhile, Akt, p38MAPK, and Erk1/2 showed a biphasic behavior (**C,D,** and **E**), which is also evident, but less prominent in Btk (**B**).

Anti-IgM stimulated PBMC (similar time points as described before) were assayed for phosphorylation of proteins associated with the BCR signaling pathways (Syk-Y352, Btk-Y551, Akt-S473, p38MAPK-T180/Y182, and Erk1/2-T202/Y204) in two separate panels. These proteins allow the simultaneous evaluation of the initiation (Syk), propagation (Btk, Akt), and integration signaling units (p38MAPK-T180/Y182 and Erk1/2-T202/Y204). Furthermore, the selected proteins can also provide some information regarding whether the PLCγ2, PI3K, or both BCR-associated signaling pathways are activated. All evaluated proteins showed enhanced phosphorylation in Um B cells when compared to naïve B cells (Figures 5A–E). As described above, Syk, which represents a protein from the initiation signaling subunit, showed higher phosphorylation in Um than in naïve B cells (Figure 5A). Interestingly, the phosphorylation peak was observed at 5 min, followed by a slow dephosphorylation slope (Figures 4B, 5A). Among proteins of the integration subunit, Btk and Akt were also evaluated. Naïve and Um cells showed an early peak of Btk phosphorylation (Y551) 1–2 min after stimulation, followed by a plateau established around minute 10 which persisted until the last time point collected (30 min). Akt was phosphorylated in naïve and Um cells; however, it was more prominent in the latter cells (Figure 5C). Interestingly, two phosphorylation peaks were identified, one between minutes 5–8 and a second one at 15–20 min. Following the second peak, pAkt-S473 levels returned to the resting state in both B cell populations. Among the proteins of the integration signaling subunit, p38MAPK (T180/Y182) and Erk1/2 (T202/Y204) were evaluated (Figures 5D,E). Phosphorylation of both proteins was identified in Um, but not in naive B cells. Furthermore, phosphorylation of these proteins also showed a biphasic phase with peaks at 2–3 and 8–10 min.

### Inhibition of phosphatases by H_2_O_2_ in naïve and Um B cells

**Figure 6:**
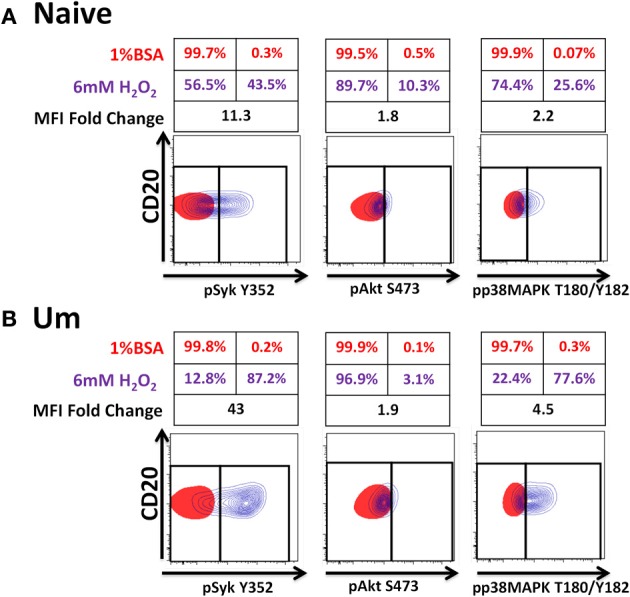
**Phosphatases in Um B cells are more susceptible to inhibition by H_2_O_2_.** PBMC were treated with 6 mM of H_2_O_2_ and phosphorylation of proteins associated with the BCR were measured in naïve and Um B cells (**A** and **B**) after 5 min of stimulation at 37°C. Displayed are overlaps of unstimulated (1% BSA) (red) and stimulated (H_2_O_2_ without anti-IgM) (purple) naïve B cells **(A)**. Similar data display was used for Um B cells **(B)**. Shown are percentages (red letter/numbers = negative controls; purple letter/numbers = H_2_O_2_ stimulation) as well as fold changes in mean fluorescence intensity (MFI) of stimulated compared to unstimulated cells. Um cells showed enhanced phosphorylation of Syk and p38MAPK **(B)**, compared to naïve B cells **(A)**. Akt phosphorylation was similar in both cell populations (**A** and **B**).

Initiation of antigen receptor signaling not only requires activation of kinases; most importantly, it requires inhibition of phosphatases (e.g., Phospho-Tyrosine-Phosphatases), which have 100–1000-times higher turnover rate than kinases. H_2_O_2_ is a universal phosphatase inhibitor, which at high doses induces phosphorylation of proteins associated with the BCR-signaling pathway mimicking antigen stimulation (Reth, 2002; Singh et al., 2005; Tonks, 2005). To determine if phosphatases of naïve and Um B cells were equally susceptible to inhibition by H_2_O_2_ and if inhibition of these proteins contributed to the enhanced phosphorylation detected in Um B cells, PBMC were treated with 6 mM H_2_O_2_ (in the absence of anti-IgM) or 1% BSA (negative control of stimulation) (5 min; 37°C). This dose of H_2_O_2_ was chosen based on previous reports from Irish et al. (2006) and our own titration experiments (not shown), which demonstrated that doses lower than 5 mM did not inhibit phosphatases to the point of inducing spontaneous phosphorylation of proteins associated with the BCR. Phosphorylation of Syk, Akt, and p38MAPK was subsequently evaluated and detected only in cells stimulated with H_2_O_2_ (Figures 6A,B). Syk and p38MAPK phosphorylation in Um was more intense than in naïve B cells (percentage and fold changes over unstimulated controls) (Figures 6A,B). Interestingly, phosphorylation of Akt was similar in both populations.

### Activation of BCR-signaling pathways in Sm CD27+ and Sm CD27− following BCR stimulation and inhibition of phosphatases by H_2_O_2_

**Figure 7:**
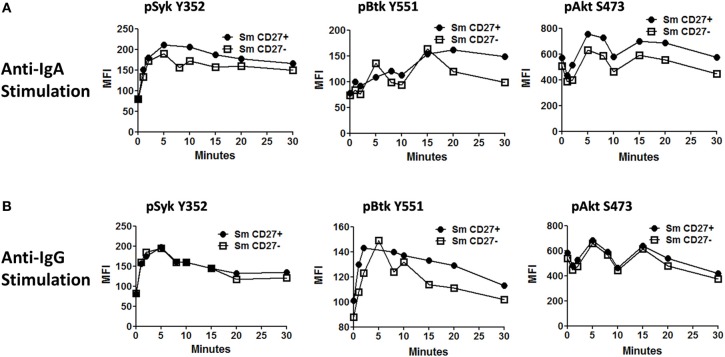
**Time course of phosphorylation of BCR-associated signaling proteins in Sm CD27+ and Sm CD27− B cells following anti-IgA and anti-IgG stimulation.** Anti-IgA and anti-IgG stimulated PBMC were assayed for phosphorylation of Syk, Btk, and Akt (**A** and **B**, respectively) at different time points (1, 2, 5, 8, 10, 15, 20, and 30 min).

**Figure 8:**
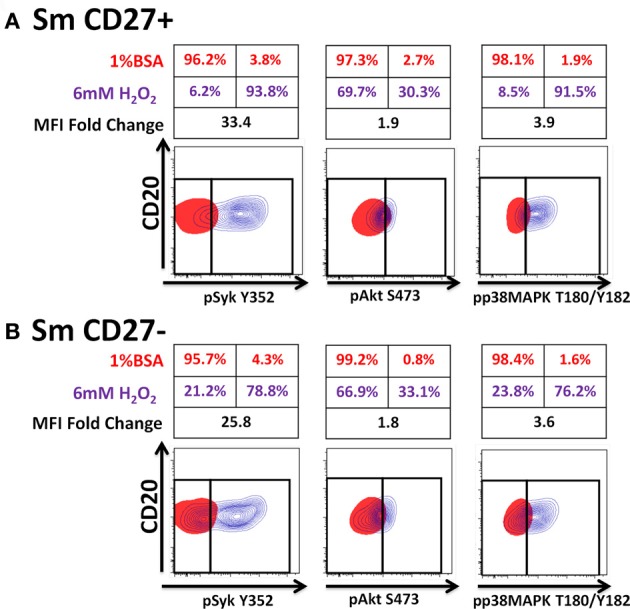
**Phosphatases in Sm CD27+ and Sm CD27− B cells are similarly susceptible to inhibition by H_2_O_2_.** PBMC were treated with 6 mM of H_2_O_2_ phosphorylation of proteins associated with the BCR were detected in both, naïve and Um B cells (**A** and **B**) after 5 min stimulation at 37°C. Displayed are overlaps of unstimulated (1% BSA) (red) and 6 mM of H_2_O_2_ stimulated (without anti-IgM) (purple) naïve and Um B-cells. Percentage as well as fold changes in MFI (as compared to unstimulated) are shown. Um cells showed enhanced phosphorylation of Syk and p38MAPK **(B)**, compared to naïve B cells **(A)**. Akt phosphorylation was similar in both cell populations (**A** and **B**).

**Figure A2:**
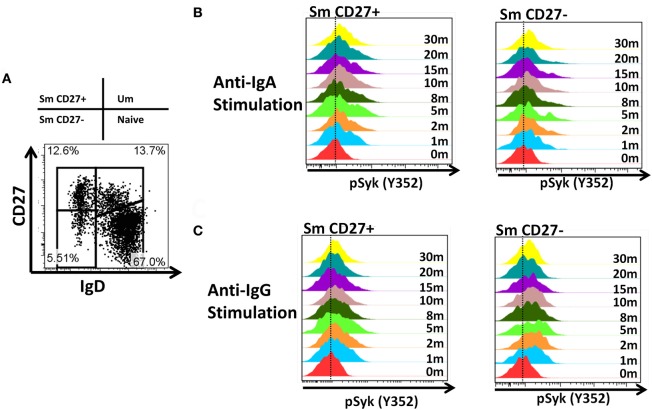
**Time course of phosphorylation of pSyk in Sm CD27+ and Sm CD27− B cells following anti-IgA and anti-IgG stimulation.** PBMC from healthy volunteers were stimulated with either anti-IgA or anti-IgG and phosphorylation of Syk was evaluated at different time points (1, 2, 5, 8, 10, 15, 20, and 30 min) in Sm CD27+ and Sm CD27− B cell populations **(A)**. Samples were fluorescently barcoded for multiplexing. Displayed are detailed histograms of pSyk following anti-IgA **(B)** and -IgG **(C)** at each time point.

Anti-IgA and anti-IgG stimulated PBMC were evaluated for phosphorylation of proteins associated with the BCR (Figures 7, A2) in Sm CD27+ and Sm CD27− B cells. Syk phosphorylation was similar between Sm CD27+ and CD27− B cells following stimulation with anti-IgA (Figure 7A—left panel and Figure A2B); the phosphorylation peak was detected between 5–10 minutes. Btk phosphorylation induced by anti-IgA increased slowly in Sm CD27+ and Sm CD27− cells, showing a late peak at 15 min. A quicker dephosphorylation in Sm CD27− B cells was recorded (Figure 7A—center panel). Anti-IgA induced slightly higher phosphorylation of Akt in Sm CD27+ cells than Sm CD27− cells. However, the phosphorylation/dephosphorylation patterns were similar in both populations (Figure 7B—right panel). In contrast, anti-IgG stimulation induced similar Syk, Btk, and Akt phosphorylation intensity and kinetics in both Sm CD27+ and Sm CD27− B cells (Figure 7B and Figure A2C). Phosphorylation peaks of Syk and Btk were observed at 5 min (Figure 7B—left and center panels), while Akt showed a biphasic phosphorylation peak (5 and 15 min) (Figure 7B—right panel). Phosphorylation of p38MAPK and Erk1/2 were also evaluated in Sm CD27+ and Sm CD27− B cells following anti-IgA and -IgG stimulation; however, no phosphorylation was detected in our assay. Finally, inhibition of phosphatases in Sm CD27+ and Sm CD27− B cells was also evaluated using 6 mM H_2_O_2_ as described above. The results showed that Akt and p38MAPK were similarly phosphorylated (Figures 8A,B). Although slight differences were detected in Syk phosphorylation (Figures 8A,B, left panels) these changes were not as intense as those detected between naïve and Um B cells (Figures 6A,B, left panels).

### Bacteria interaction and signaling induced in naïve and Um B cells

**Figure 9:**
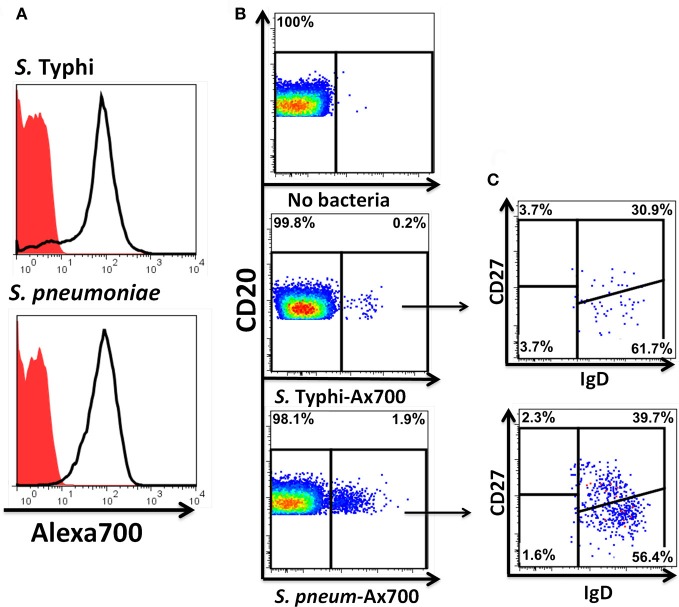
**Fluorescently labeled bacteria bind preferentially to naïve and Um B cells.** Heat-killed *S*. Typhi (gram negative) and *S. pneumoniae* (encapsulated gram positive) bacteria were fluorescently labeled with Alexa700 **(A)**. Unstained bacteria (red closed histograms) and stained bacteria (continuous lines) are shown in panel **A**. PBMC were incubated with labeled bacteria at a MOI of 100:1 for 10 min at 37°C and the population of B cells recognizing the bacteria identified **(B)**. A higher percentage of B cells recognizing *S. pneumoniae* than *S*. Typhi **(B)** was observed. A higher proportion of naïve than Um B-cell subpopulations was found to interact with the bacteria **(C)**. **B** and **C** show data observed in a representative volunteer.

**Figure 10:**
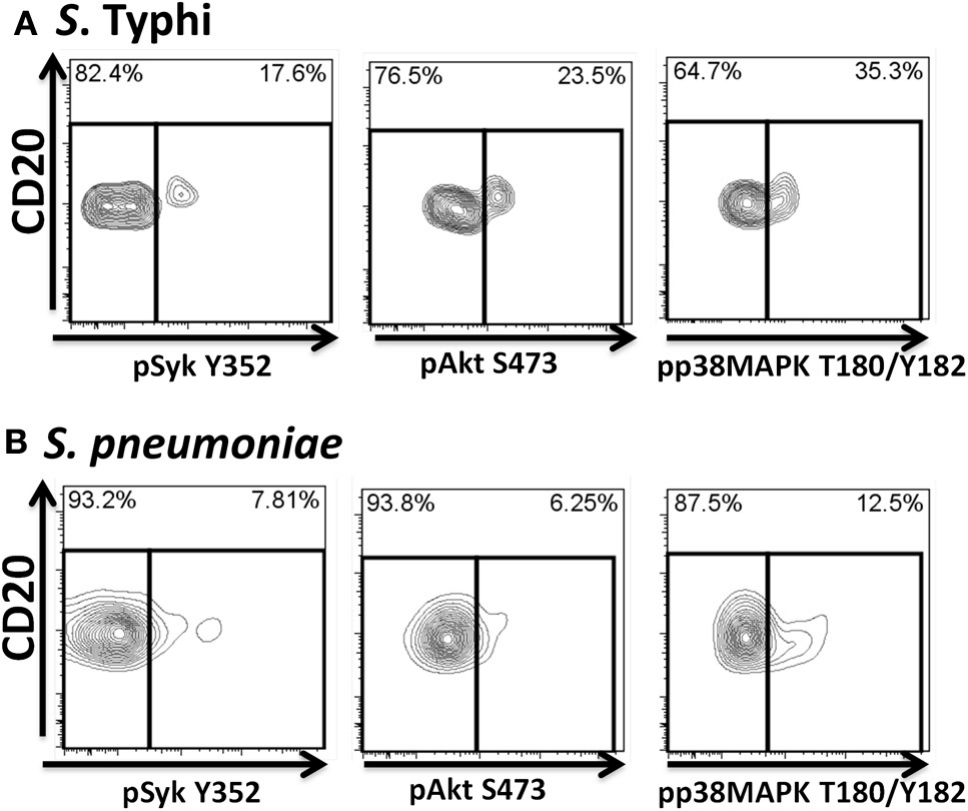
**Bacteria-induced cell signaling in Um B cells.** B cells interacting with fluorescently labeled bacteria were assayed for phosphorylation of signaling proteins (Syk, Akt, and p38MAPK). Shown are phosphorylation patterns in Um B cells from a representative volunteer following stimulation with *S*. Typhi **(A)** or *S. pneumoniae* **(B)** at the 10 min time point.

**Figure 11:**
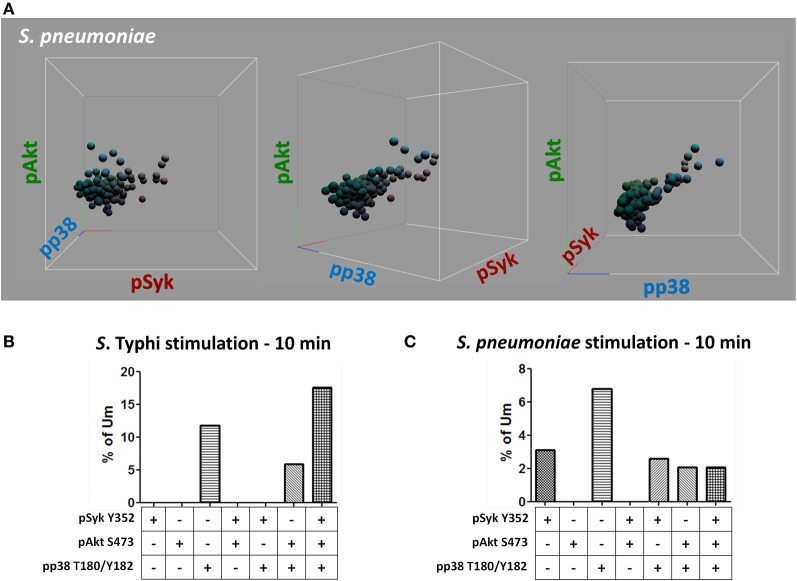
**Multiphosphorylated Um B cells.** Whether single or multiple proteins were phosphorylated simultaneously **(A)** was evaluated by gating on Um cells which bound *S*. Typhi or *S. pneumoniae* (**B** and **C**, respectively). Multiple tri-dimensional views (rotated on the X axis) from a representative volunteer are displayed (stimulation with *S. pneumoniae* for 10 min) **(A)**. Spheres represent individual cells and the colors of the spheres are the result of combination of primary colors at the origin of each axis [x axis: red (pSyk); y axis: blue (pAkt); and z axis: green (pp38MAPK)] **(A)**. Percentage of Um B cells that showed, one, two, or three proteins phosphorylated simultaneously following stimulation with *S*. Typhi or *S*. pneumoniae (**B** and **C**, respectively).

Heat-killed *S*. Typhi (gram negative) and *S. pneumoniae* (encapsulated gram positive) fluorescently labeled with Alexa700 (Figure 9A) were used to stimulate PBMC (MOI 100 to 1) (37°C; 10 min) from healthy volunteers. These bacteria were selected because they have different antigenic structures and it was of interest to determine whether they could induce different signaling cascades on B cells. Relatively small populations of B cells interacting with *S*. Typhi (0.23%) and *S. Pneumoniae* (1.9%) were identified (Figure 9B) and these cells corresponded mainly to naïve and Um B cells (Figure 9C). A higher percentage of naïve than Um B cells (ratio 2:1) interacted with both bacteria (Figure 9C). Phosphorylation of various B cells signaling proteins (Syk, Akt, and p38MAPK) was then assayed in these subpopulations (Figures 10A,B). Even though a higher percentage of naïve B cells interacted with both bacteria, these cells did not show phosphorylation of the assayed proteins (data not shown). On the other hand, phosphorylation of Syk, Akt, and p38MAPK was induced in Um B cells by both *S*. Typhi and *S. pneumoniae* (Figures 10A,B). The percentage of phosphorylated cells for each protein was consistently higher in cells stimulated with *S*. Typhi than with *S. pneumoniae* (Figures 10A,B). Finally, to better demonstrate the complexity of the signaling responses induced in Um B cells by bacteria we used this novel phosphoflow technique to determine the percentage of cells that showed single, double, or triple phosphorylation of the signaling proteins assayed, i.e., whether they are multi-phosphorylated (Figure 11). We observed, for the first time, the presence of multi-phosphorylated cells and determined that the signaling responses were different among cells stimulated with *S*. Typhi and *S. pneumoniae*. *S*. Typhi-stimulated cells showed a higher percentage of Um B cells that phosphorylated the three proteins (18%) assayed, followed by cells that showed only p38MAPK phosphorylation (12%) and finally 5% showing Akt and p38MAPK phosphorylation (Figure 11B). On the other hand, the percentages of Um B cells that phosphorylated proteins following *S. pneumoniae* stimulation were overall smaller. Um B cells that phosphorylated only p38MAPK (7%) were the predominant ones. Around 2% of Um cells showed single (Syk), double (Syk, p38MAPK, or Akt, p38MAPK) and triple (Syk, Akt, p38MAPK) phosphorylated proteins (Figure 11C).

## Discussion

Traditionally, signaling pathways in B cells have been studied using established cells lines (e.g., DT40) or cell models transfected with various signaling proteins associated with the BCR (e.g., drosophila S2 cells). These studies have been complemented by the use of mouse models (e.g., knock-outs and knock-ins) and have relied heavily in western-blotting and ELISA techniques (Takata et al., 1995; Rolli et al., 2002; Srinivasan et al., 2009; Young et al., 2009). Despite the usefulness of these models/techniques and the invaluable data generated, they present various limitations including the large number of cells required for experimental procedures, inability to study low frequency cell populations, inability to study the phosphorylation of proteins involved in signaling pathways at the single cell level, and in some cases the difficulty of translating the results from animal models to humans. The advent of multi-chromatic FC and availability of monoclonal antibodies for specific phospho-epitopes has allowed the development of phosphoflow, a novel technique that permits the evaluation of multiple phosphorylated epitopes simultaneously at the single cell level in heterogeneous cell mixtures. We have used this powerful emerging technique to study signaling pathways of activation in naïve and B_M_ cell populations, in human primary cells, following stimulation of the BCR. Furthermore, the use of fluorescently labeled bacteria allowed the identification of B cell subpopulations that interact with bacteria and provided a glimpse at the complex and diverse signaling pathways activated by gram positive and gram negative bacteria in primary human cells. These studies have important implications for vaccine development since they provide a novel platform that, within a few minutes of *in vitro* exposure to specific antigens, might identify the signaling pathways elicited in appropriate B subsets that may lead to the induction of short and long-lived antibody-producing plasmablasts/plasmocytes and long-term B memory cells.

Immunological memory is one of the key factors in the development of long-lasting immune responses to specific antigens (Levine and Sztein, 2004; Lanzavecchia et al., 2006; Simon et al., 2009, 2011; El-Kamary et al., 2010; Ramirez et al., 2012; Wahid et al., 2012). In the case of the B cell compartment, memory is generally accepted to be provided by two subpopulations: plasma cells and B_M_ cells (Sanz et al., 2008). Plasma cells, which produce antibodies, represent an effector memory cell type. Meanwhile, B_M_ cells represent a central memory cell type due to their ability to generate and replenish the plasma cell compartment. However, this traditional view is changing since important effector and regulatory functions that might be played by central B_M_ cells (e.g., cytokine production, antigen presentation, DC regulation) have been described (Shimamura et al., 1982; Blair et al., 2010; De Wit et al., 2010; Lund and Randall, 2010; Biragyn and Lee Chang, 2012). Our knowledge of the early events in B cell activation in naïve and memory subpopulations is limited. Understanding how various ligands activate different signaling pathways is dramatically important since these events will ultimately determine the cell's fate [antibody or cytokine production (or both), antigen presentation, apoptosis, etc.]. Four naïve and B_M_ cell populations are defined by IgD and CD27 surface markers (naïve, Um, Sm CD27+, and Sm CD27−) (Figure 1A). Expression of additional surface markers (e.g., class switched Ig, CD21, CD23, CD1c) as well as the level of mutations in the Ig V genes for these populations have been reported by various groups (Weller et al., 2004; Fecteau et al., 2006; Tangye and Good, 2007; Pauli et al., 2011; Wu et al., 2011). However, to our knowledge, no study has reported similarities/differences in signaling pathways associated with BCR activation in these B cell subsets in primary human cells. These early events are critical for determining the maturation and differentiation of the activated B cells. To study BCR-associated signaling pathways, we modified a phosphoflow technique previously published (Krutzik and Nolan, 2003; Perez and Nolan, 2006; Schulz et al., 2007; Krutzik et al., 2011b) to accommodate the expression of molecules that allowed appropriate resolution of naïve and B_M_ cell population defined by IgD and CD27 (Figure A1). Furthermore, the phosphoflow assay was coupled with FCB for multiplexing (Figure 4A) (Krutzik and Nolan, 2006; Krutzik et al., 2011a). We demonstrated that Syk is phosphorylated in naïve and B_M_ cells only when stimulated with antibodies directed to Igs expressed by the specific sub-population (assay specificity). For example, stimulation of PBMC with anti-IgM led to Syk phosphorylation only in naïve and Um B cells, which are the main populations expressing IgM (Figure 3A). IgM also induced a small peak in Sm CD27+ cells in some volunteers (Figure 3A), which corresponded to a small population of IgM expressing cells within Sm CD27+ B cells (Figure 1D). Conversely, PBMC stimulated either with anti-IgA or -IgG showed phosphorylation of Syk only in Sm CD27+ and Sm CD27− populations (Figures 3B,C). However, since IgA and IgG are expressed in about half of Sm CD27+ and Sm CD27− (Figure 1D) a dual peak (positive and negative) was shown in each case (Figures 3B,C). These results demonstrated the specificity of the assay and the feasibility of this approach to explore phosphorylation at the single cell level in individual subsets based on surface expression of defined molecules.

Among the B cell populations defined by IgD and CD27, naïve and Um B cells co-express IgM and IgD; however, the latter showed a higher frequency of IgM (Figure 2). This led to the investigation of whether Um B cells were more susceptible to Syk phosphorylation (and therefore activation) than naïve B cells, when stimulated with anti-IgM antibodies. As expected Um B cells showed a stronger phosphorylation of Syk at each time point evaluated (Figures 4B,C). Furthermore, Um B cells had a higher total amount of Syk protein (phosphorylated and non-phosphorylated) (Figure 4D) and their phosphatases were more susceptible to inhibition by H_2_O_2_ (Figure 6). Taken together, these data suggest that Um B cells have a lower activation threshold than naïve B cells. Subsequent experiments demonstrated that the differences in Syk phosphorylation between naïve and Um cells were maintained in the downstream signaling proteins evaluated (Akt, p38MAPK, and Erk1/2), since in all instances, except for Btk, Um cells showed different degrees of higher phosphorylation (Figure 5). Given that, naïve and Um B cells phosphorylated Btk and Akt, the PI3K, and survival pathways appear to be activated in both B cell populations. However, p38MAPK and Erk1/2 were phosphorylated only in Um cells, which suggest that the PLCγ2 pathway was activated only in these cells.

Um B cells, also known as memory IgM cells, are a controversial population (Tangye and Good, 2007; Seifert and Küppers, 2009). These cells were initially classified as a type of memory cells due to the expression of CD27. However, these cells are still present in patients with hyper-IgM syndrome (HIGMS), the immunodeficiency X-linked lymphoproliferative disease (XLP) and in common variable immunodeficiency (CVID). HIGMS is the result of mutations in the *CD40* or CD40 ligand (*CD40L*) genes. XLP results from mutations in the *SH2D1A* gene, while CVID from mutations in *ICOS*. All these disorders result in the absence of B_M_ and patients do not undergo Ig isotype switching to produce IgG, IgA, and IgE in response to T cell-dependent antigens. Additionally, these patients have alterations in germinal centers (Facchetti et al., 1995; Gulino and Notarangelo, 2003; Ma et al., 2005, 2006; Warnatz et al., 2006). Finally, various recent reports have shown that Um B cells are critical for protection against T cell-independent antigens [e.g., encapsulated bacteria such as *S. pneumoniae* and *N. gonorrhea* (Zandvoort and Timens, 2002; Kruetzmann et al., 2003; So et al., 2012)]. Therefore, the presence of Um in patients with defects that lead to the absence of B_M_ (HIGMS, XLP, and CIVD), their importance in protection against T cell-independent antigens and maturation through germinal center-independent pathways, suggests that these cells are not a true B_M_ population, but rather a separate population of innate or naïve B cell (Weller et al., 2004). Based on this and other evidence, Um B cells have been proposed to be the human equivalent of mice's marginal zone B cells, which play an important role in responses to T cell-independent antigens and have a lower activation threshold (Zandvoort and Timens, 2002; Weller et al., 2004; Lopes-Carvalho et al., 2005). Our data support this hypothesis since compared to naïve, Um B cells had a lower activation threshold, which dependent at least on three factors: (1) higher IgM receptors of the surface, (2) higher total levels of the Syk protein, and (3) enhanced susceptibility to phosphatase inhibition. Other factors, such as the absence or presence, or different expression levels, of co-receptors, can also contribute to further lower the activation threshold and will be explored in future studies.

PBMC stimulated with anti-IgA and -IgG showed phosphorylation of Syk in Sm CD27+ and Sm CD27− B cells, at similar rates with both stimulants suggesting that these populations have similar activation thresholds. Furthermore, as previously discussed, unlike naïve and Um B cells, Sm CD27+ and Sm CD27− B cells showed a positive and negative peak since approximately 50% of the cells within these populations expressed one or the other Ig. Additionally, the intensity of Syk phosphorylation was much lower than that induced by anti-IgM in Um B cells (Figure 5A), which is not surprising since only approximately half of the cells were expected to respond to stimulation. Syk phosphorylation activated downstream signaling proteins and overall, Sm CD27+ and Sm CD27− B cells responded equally to IgG and IgA stimulation, with only minor differences. Most notably, Akt showed a slow phosphorylation slope, with a peak at 15 min, when cells were stimulated with anti-IgA (Figure 7A, central panel). Meanwhile, anti-IgG induced a quick response (peak at 5 min) followed by a shallow dephosphorylation slope (Figure 7B, central panel). Interestingly, Akt phosphorylation was bi-phasic, with peaks at 5 and 15 min, in Sm CD27+ and Sm CD27− cells. A slightly higher phosphorylation intensity of Akt was detected when anti-IgA was used (Figures 7A right panel vs. 7B right panel). These data suggest that both stimulants-induced activation of the survival pathway in these cells. Neither anti-IgA nor -IgG, induced detectable levels of phosphorylated p38MAPK nor Erk1/2 (not shown), suggesting that the PLCγ2 pathway was neither activated in Sm CD27+ nor in Sm CD27− B cells. Overall, Sm CD27+ and Sm CD27− had strong similarities in the signaling pathways activated, as well as in the activation threshold. Additional evidence to further support the notion that both populations have similar activation thresholds comes from H_2_O_2_ stimulation experiments that resulted in similar phosphatase inhibition, with the exception of Syk. However, no Syk phosphorylation differences were identified when anti-IgA or -IgG were used. Future experiments using other ligands as well as co-receptor stimulation might provide data to determine if the differences in the inhibition of phosphatases that control Syk are important for other stimulants. Finally, a caveat in interpreting these experiments is that they might not provide enough resolution for studying the phosphorylation status of some proteins in Sm CD27+ and Sm CD27− B cell populations, due the mixed surface Ig expression. In future experiments, modification to the staining techniques to allow selection of cells expressing only IgA or IgG most likely will reveal more details of the signaling pathways in these cells and will more finely define the role of these populations in the development of immune responses to pathogenic agents or vaccination.

Various reports have shown a prominent role of Um B cells in protection against encapsulated bacteria. The evidence comes from the enhanced susceptibility that infants, splenectomized patients and people affected by CVID have for infections by *S. pneumonia* (Kruetzmann et al., 2003). In all these cases Um B cells are undetectable in blood. Furthermore, various studies have shown that pneumococcal polysaccharide (PS) vaccines are poorly immunogenic in young children (infants). As the immune system in children matures, the immune responses to pneumococcal PS vaccination improve, which correlate with an increase in the frequency of Um cells in peripheral blood (Kruetzmann et al., 2003). Recent reports have shown that Um cells also play an important role in *N. gonorrheae* (another encapsulated bacterium) infections (So et al., 2012). To determine if exposure to encapsulated bacteria resulted in a preferential activation of Um cells, PBMC from healthy volunteers were exposed to Alexa 700-labeled heat-killed *S. pneumoniae* (Gram +) and *S*. Typhi (Gram −) bacteria. Surprisingly, *S. pneumoniae* interacted with both naïve and Um cells. Of note, a higher percentage of naïve and not Um B cells (2:1 ratio) interacted with these bacteria (Figure 9B—bottom panel). These results were similar in PBMC exposed to *S*. Typhi; however, a lower percentage of B cells interacted with these bacteria (Figure 9B—middle panel). Of note, despite the higher percentage of naïve B cells interacting with both bacteria, no phosphorylation of the assayed proteins (Syk, Akt, and p38MAPK) was detected in this population (data not shown). On the other hand, Um B cells that interacted with both bacteria showed phosphorylation (Figures 10A,B). To study the patterns of B cell phosphorylation in greater detail, we used a unique feature of phosphoflow, i.e., its capacity to explore the activation of multiple signaling pathways simultaneously at the single cell level. Analogous to the term in widespread use to signify the production of multiple cytokines by single cells, we would like to propose the use of the term “multiphosphorylated cells” to indicate the presence of more than one signaling pathway activated in a single cell. Using this technology, we observed that the multi-phosphorylated cell patterns induced by *S. pneumoniae* and *S*. Typhi were dramatically different (Figures 11B,C). This is not surprising since these bacteria have different antigenic structures. The induction of phosphorylation in Um B cells might be due to the lower threshold needed to activate these cells. Furthermore, this supports the idea that Um B cells are indeed a type of innate B cell. The lack of phosphorylation in naïve B cells does not necessarily indicate that these cells were not responsive to the stimuli. It is possible that these cells interact with the bacteria by other receptors which induce signaling through pathways not evaluated in the current study. Moreover, these results might reflect the fact that the signaling pathways in naïve B cells are more tightly regulated and that additional and/or stronger signals are required for activation. The nature of those additional signals (e.g., second messengers such as H_2_O_2_ to lower the activation threshold, cytokines, interaction with other cells of the immune system) remains to be explored.

Among phosphatase inhibitors, H_2_O_2_ has gained attention lately because BCR-ligand stimulation induces H_2_O_2_ production and cells use this molecule as a second messenger for signal transduction and amplification (Reth, 2002). Furthermore, various studies have shown that stimulation of B cells through other receptors might also induce H_2_O_2_; for example, LPS, which binds to toll-like receptors (TLRs) is a well know inducer of reactive oxygen species (including H_2_O_2_) (Deleo et al., 1998; Kawahara et al., 2001). Moreover, *in vivo* during the development of an immune response, B cells most likely receive cell signals by different receptors simultaneously (e.g., BCR, CD40, CD19, TLRs, etc.). Thus, co-receptor stimulation can lead to enhanced H_2_O_2_ production that ultimately will reduce the threshold for B cell activation. Bacteria have a conglomerate of antigens on the surface; therefore, stimulation of PBMC with *S. pneumoniae* and *S*. Typhi is likely to stimulate several receptors on B cells. The latter consideration, together with the lower activation threshold of Um B cells as suggested by the higher total Syk levels and enhanced susceptibility of phosphatases to be inhibited, might all have contributed to enhance Um activation.

A more detailed account of the receptors stimulated by bacteria remains to be explored. However, it is reasonable to speculate that TLR4 and CD14 receptors are involved in interaction with LPS from *S*. Typhi (Ganley-Leal et al., 2010). TLR4 expression has been reported to be present at low levels in B cells from healthy volunteers (Ganley-Leal et al., 2010); however, which B cell subpopulation(s) express these receptors has not been described. Given the results obtained in our experiments we speculate that Um B cells are part of a subset of B cells expressing this receptor. LPS-induced signaling through TLR4 requires CD14 and this receptor also has been reported to be expressed in B cells (Ziegler-Heitbrock et al., 1994). Therefore, the identification of cells that phosphorylated only p38MAPK (Figure 11B) suggest that these Um B cells not only express TLR4 and CD14, but also that they are functional. Another innate receptor to consider is TLR5, which has shown to recognize *Salmonella* flagellin. Similar to TLR4, TLR5 can lead to phosphorylation of p38MAPK. Co-phosphorylation of Syk/Akt/p38MAPK or Akt/p38MAPK suggests that BCR was involved in the phosphorylation of p38MAPK. On the other hand, *S. pneumoniae* is known to contain ligands for TLR2, TLR4, and TLR9. Among the latter, the first two can lead to p38MAPK phosphorylation alone, which was the higher population identified among Um B cells (Figure 11C). All other populations identified with phosphorylation of Syk, Syk/p38MAPK, Akt/p38MAPK, or Syk/Akt/p38MAPK indicate that BCR interaction was involved at some level. These results show the complexity of interaction of bacteria with Um B cells and suggest that various receptors are involved in the initial interaction with these microorganisms. More detailed studies are required to further dissect these complex interactions.

In conclusion, phosphoflow is proving to be an important tool to understand basic mechanisms in the early process of B cell activation in primary human cells. In the current study, differences between BCR-dependent signaling pathways among naïve and Um cells were highlighted. Future studies will address the role of co-receptor stimulation in BCR activation (e.g., CD40, CD19/CD21) and how these cooperate to lower/increase the activation threshold, activate different pathways or induce different responses. The current study provided evidence that in healthy volunteers, naïve and Um B cells are the main subpopulations interacting with bacteria within minutes of exposure and that different bacteria induce different signaling profiles in Um B cells. Future studies will address if these signaling profiles are comparable among groups of bacteria (e.g., conserved responses to gram positive/negative bacteria) and other microorganism (virus, parasites, etc.). Phosphoflow holds great potential for future studies in vaccine development, for example could lead to the identification of signaling profiles in good/poor responders and predict if booster immunizations would be necessary weeks before antibody titers could be detected. Additionally, in the development vaccines to PS could lead to the identification of subpopulations of B cells that recognize conjugated and unconjugated vaccines and determine if a particular carrier protein will lead, or not, to the development of T cell memory. Furthermore, the frequency and signaling profiles of vaccine/pathogen specific B cells (Um cells, as well as Sm CD27+ and Sm CD27− populations) can be assayed in specimens collected from immunized subjects or exposed to natural infection. In summary, phosphoflow is a powerful technique that can be useful not only to study basic aspects of cell B cell biology, but also in studies in translational and clinical medicine to forecast responses to immunization and help improve vaccine design by suggesting which B cell populations need to be stimulated in order to achieve appropriate immune responses.

### Conflict of interest statement

The authors declare that the research was conducted in the absence of any commercial or financial relationships that could be construed as a potential conflict of interest.

## Appendix

### Development of panels for resolving B cell naïve and memory subpopulations by phosphoflow

One of the limiting factors in developing phosphoflow assays is the availability of antibodies that recognize target epitopes after cell fixation (2% PFA) and permeabilization (>80% methanol). Exposure of cells to these treatments destroys or dramatically alters most of the common epitopes (e.g., CD19). A limited number of clones that allow appropriate resolution of the population are available (Cytobank.org) (e.g., CD27-L128, CD20-H1). Despite this, various limitations remain since antibody clones for various common epitopes have either not been tested or available; among these epitopes are those for surface Ig isotypes (e.g., IgD, IgM, etc.). In order to develop phosphoflow panels to study naïve and memory B cell populations, a mixed staining technique was used. This technique involved staining of selected epitopes immediately after cell fixation (PFA), but before permeabilization (methanol). Therefore, antibodies needed to fulfill two characteristics: (1) appropriate epitope recognition after treatment of the cells with PFA and (2) resistance of the conjugated fluorochrome to methanol permeabilization. Various monoclonal antibodies to human IgD, IgM, IgG, and IgA were tested unsuccessfully using the mixed technique (PFA/MeOH) (data not shown). However, polyclonal sera [goat F (ab')2] anti-human-IgD and –IgM conjugated to FITC allowed resolution of naïve and memory B cell populations similar to normal surface staining (Figures A1A,B) (see “Materials and Methods” for detailed description of staining technique).
